# Extracting medication information from unstructured public health data: a demonstration on data from population-based and tertiary-based samples

**DOI:** 10.1186/s12874-020-01131-7

**Published:** 2020-10-15

**Authors:** Robert Chen, Joyce C. Ho, Jin-Mann S. Lin

**Affiliations:** 1 Chronic Viral Diseases Branch, Division of High-Consequence Pathogens and Pathology, National Center for Emerging and Zoonotic Infectious Diseases, Centers for Disease Control and Prevention, 1600 Clifton Rd. NE, Mailstop H24-12, Atlanta, GA 30329 USA; 2 School of Medicine, Emory University, 1648 Pierce Dr NE, Atlanta, GA 30307 USA; 3 Department of Computer Science, Emory University, 400 Dowman Drive, Atlanta, GA 30322 USA

**Keywords:** Automation, Data extraction, Unstructured data, Medication, Natural language processing, Mylagic encephalomyelitis/chronic fatigue syndrome (ME/CFS), Co-morbidity, Population, Tertiary

## Abstract

**Background:**

Unstructured data from clinical epidemiological studies can be valuable and easy to obtain. However, it requires further extraction and processing for data analysis. Doing this manually is labor-intensive, slow and subject to error. In this study, we propose an automation framework for extracting and processing unstructured data.

**Methods:**

The proposed automation framework consisted of two natural language processing (NLP) based tools for unstructured text data for medications and reasons for medication use. We first checked spelling using a spell-check program trained on publicly available knowledge sources and then applied NLP techniques. We mapped medication names into generic names using vocabulary from publicly available knowledge sources. We used WHO’s Anatomical Therapeutic Chemical (ATC) classification system to map generic medication names to medication classes. We processed the reasons for medication with the Lancaster stemmer method and then grouped and mapped to disease classes based on organ systems. Finally, we demonstrated this automation framework on two data sources for Mylagic Encephalomyelitis/ Chronic Fatigue Syndrome (ME/CFS): tertiary-based (*n* = 378) and population-based (*n* = 664) samples.

**Results:**

A total of 8681 raw medication records were used for this demonstration. The 1266 distinct medication names (omitting supplements) were condensed to 89 ATC classification system categories. The 1432 distinct raw reasons for medication use were condensed to 65 categories via NLP. Compared to completion of the entire process manually, our automation process reduced the number of the terms requiring manual labor for mapping by 84.4% for medications and 59.4% for reasons for medication use. Additionally, this process improved the precision of the mapped results.

**Conclusions:**

Our automation framework demonstrates the usefulness of NLP strategies even when there is no established mapping database. For a less established database (e.g., reasons for medication use), the method is easily modifiable as new knowledge sources for mapping are introduced. The capability to condense large features into interpretable ones will be valuable for subsequent analytical studies involving techniques such as machine learning and data mining.

## Background

Clinical epidemiological studies commonly involve collection of data via surveys administered to patients and providers which may be useful for analytical studies such as identification of medication treatment patterns, discovery of disease subtypes, or analysis of outcome differences. Extraction of relevant valuable information on medication usage is difficult because it is often stored in free text from these epidemiological surveys. For example, relevant aspects of medication usage such as medication names, dosage, mode, frequency and reasons for taking medications are often stored in free text and are difficult to extract for subsequent data analytics efforts. Research studies would typically resort to cumbersome and time intensive manual data extraction performed by certified data extractors who are trained to read unstructured text and assign labels to them. The protocol performed by a certified extractor would typically involve extraction of a concept followed by manual curation.

Due to the unstructured nature of reporting medications and co-morbid conditions as free text, data from epidemiological studies may be difficult to readily use in data analysis. For example, a medication may be stored with over 10 different representations as unstructured text, either due to misspelling of the medication name, usage of a generic or brand name, or usage of an abbreviation due to his or her previous training and practices. Further, sources of inconsistency may result when a medication is recorded along with dosage information. A healthcare practitioner may make a record for the medication ibuprofen as “ibuprofen”, “Advil”, “Extra Strength Advil”, “ibuprofen 200 mg”.

Previous work has focused on extracting medication names and disease names from unstructured text in the form of medical notes. One commonly used natural language processing (NLP) framework in the medical domain is cTAKES [1], which extracts relevant medical concepts from unstructured text in clinical notes and annotates them. There are several examples of NLP for specific disease related applications, such as the Clinical Record Interactive Search Comprehensive Data Extraction (CRIS-CODE) project [2] which aims to extract relevant concepts related to severe mental illness. Although there is substantial effort in NLP for clinical notes in electronic health records, there is limited work on extraction of medical concepts and subsequent mapping into interpretable categories, from unstructured text from epidemiological studies. It should be noted that there are various drawbacks to existing techniques. It requires substantial context to infer various properties of diseases and medications for most of the aforementioned techniques in text processing. For example, cTAKES focuses on named entity extraction from free-form clinical notes and requires model training on multiple corpuses to extract concepts with adequate accuracy. In epidemiological or public health studies, the information on co-morbidities or medications is frequently unavailable or not collected and thus cannot be utilized.

It is important to group raw medication names into categories in order to make subsequent model building easier and improve interpretability. In cases where medication data are recorded in a structured format, such as in electronic health records, medications are typically recorded with identification numbers tied to a specific medication grouping mechanism such as RxNorm [3], and can thus be grouped and categorized in an automated fashion. Before medication hierarchies such as the Anatomical Therapeutic Chemical (ATC) Classification System [4], and the Medical Subject Headings Pharmacological Actions (MeSHPA) [5], it was generally difficult to perform grouping of medications into categories following a structured, standardized manner. Presently, these hierarchies can be used; however, the lack of an automated process for extracting and grouping medications stored in free text remains a key limitation. Furthermore, since epidemiological studies often collect self-reported data from study participants, medication data may include supplements that the subjects consume independently of a provider’s prescription. Thus, for many analytical studies performed on epidemiological data, it is important to exclude supplements from the analysis.

Our study aims to propose an end-to-end workflow for automatic text processing and grouping into interpretable categories of smaller dimensionality than present in the raw form, specifically for medications and the condition or symptom for which medication is used. The aggregation of concepts has traditionally been difficult and time-consuming due to the lack of automated computational frameworks for mapping specific medication names into therapeutic classes and condition or symptom names into disease classes. We extracted medication names from various ontologies including RxNorm, Drugs.com [6] and WebMD [7], and subsequently mapped the raw medication data names to higher order categories based upon WHO’s Anatomical Therapeutic Chemical (ATC) classification system [4].

We also processed the data on conditions or symptoms being treated (“reason data”) using a stemming technique and mapped stems for diseases into higher order disease categories based on distinct organ systems involved. A main motivation for grouping conditions or symptoms via NLP is that there is a lack of well-known categorical classifications other than Systematized Nomenclature of Medicine (SNOMED) [8]. A limitation of SNOMED is that it may not be specific enough for domain-specific problems such as subtyping of specific diseases. We applied our overall workflow to two datasets from studies of myalgic encephalomyelitis/chronic fatigue syndrome (ME/CFS) and successfully reduced the dimensionality of distinct medication and reason data sets into a smaller, more interpretable feature sets which may be readily used in data analytics studies.

### Objective

This study sought to develop and test an innovative automation framework based on NLP for processing and condensing unstructured medical data into interpretable feature sets for use in data-driven analysis. Specifically, the study sought to:

1. Propose and implement an automation framework that leverages NLP methods combined with known ontologies to uncover interpretable feature sets of medications and reasons for their use.
2. Demonstrate the efficacy of the automation framework on two data sources from epidemiological studies of myalgic encephalomyelitis/chronic fatigue syndrome (ME/CFS).

## Methods

We first describe the proposed automation framework for extracting medication information including medication names and reasons for medication use (conditions or symptoms). Next, we describe the steps for implementing this automation framework including extracting and processing text information for further data analysis. Finally, we briefly describe how the extracted features can be used in subsequent studies involving analysis of unstructured text.

### Automation framework

**Fig. 1:**
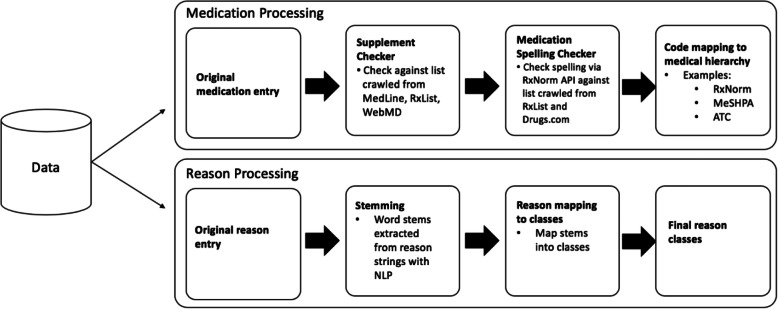
General workflow of data processing for medication and reason entries

The automation framework consists of two NLP-based tools for extracting medication information from unstructured text data including medication names and reasons for medication use. The general workflow is illustrated in Fig. 1. We process medication names, through a series of automated steps to yield drug classes where medications are grouped together because of their similarity. Similarly, we process reasons for medication use through a series of steps to yield disease groups where similar diseases are placed into higher order categories based on organ systems of involvement. An end-to-end illustration along with source code with synthetic data can be found in the Supplementary Information, Additonal file 1 (Fig. A.1).

#### Processing and mapping of medication names

Below we outline the steps we take to process and map raw medication names originally represented as unstructured text. First, we remove any medication entries that represent supplements (e.g., vitamins, minerals, etc.). Next, we apply a spell checker to correct the medication spelling. Finally, we map the “raw” drug names to drug classes according to the ATC classification system.

For extraction of supplement names, we first create an aggregated list of common supplements via a web-crawling algorithm that we applied to several existing databases: Drugs.com, an online database containing drug names and higher-level classes, RxList.com [9], a website containing drug and supplement information in which names of drugs and supplements are grouped according to classes, and the WebMD database, which contains information for common supplements. Next, we compare raw medication names against supplement names in this aggregated list in order to verify classification of supplements. For example, our method would classify medication “Vitamin C” as a supplement, while it would classify the medication “metoprolol” as a drug. For any given raw medication name, we compute its Levenshtein distance [10] against all names in the supplement dictionary. The Levensthein distance for two strings, *a* and *b,* which have length *|a|* and |*b*|, respectively, is defined as *lev*_*a*, *b*_ (*|a|*, |*b*|), where

$$ lev{}_{a,b}\left(i,j\right)=\left\{\begin{array}{cc}\max \left(i,j\right)\ & if\min \left(i,j\right)=0,\\ {}\min \left\{\begin{array}{c} lev{}_{a,b}\left(i-1,j\right)+1\kern0.50em \\ {} lev{}_{a,b}\left(i,j-1\right)+1\kern0.50em \\ {} lev{}_{a,b}\left(i-1,j-1\right)+{1}_{\left({a}_i\ne {b}_j\right)}\end{array}\right.& otherwise.\end{array}\right. $$

${1}_{\left({a}_i\ne {b}_j\right)}$ is the indicator function equal to 0 when *a*_*i*_ ≠ *b*_*j*_ and equal to 1 otherwise, and *lev*_*a*, *b*_(*i*, *j*) is the distance between the first *i* characters of *a* and the first *j* characters of *b*. let the Levenshtein ratio be accordingly defined as

$$ \frac{\left(\left|a\right|+\left|b\right|\right)- lev{}_{a,b}\left(i,j\right)\ }{\left|a\right|+\left|b\right|} $$

If the raw medication name achieves a Levensthein ratio of greater than 0.85 from any of the supplement names in the database, then we classify the medication as a drug and remove the entry from further processing. We compare the scraped drug names with the raw medication data using Levenshtein distance, following the same protocol for comparing raw medication names as was used for filtering out supplements.

In the next step, common misspellings are corrected automatically. Similar to the supplement checker, we aggregate a list of drug names crawled from RxList.com and Drugs.com. We subsequently use the aggregated list of medications to compare against raw medication names to correct misspellings.

To perform extraction from the RxNorm database, we leverage the Representational State Transfer Application Programming Interface (REST API). A REST API protocol in a web application allows for efficient transfer of data between a computer and web application hosted on a web server. We wrote scripts in the Python programming language using the Beautiful Soup package [11] in order to extract drug and supplement information from Drugs.com and WebMD. Afterwards, we combine the resulting drug and supplement names into a dictionary from which we compare the raw data.

We compare scraped drug names to the raw drug names in the unstructured data using the python-Levenshtein module, a Python package which compares similarity of two strings via Levenshtein distance [10].

If any raw drug name in the raw unstructured data matches a drug name in the drug dictionary with a Levenshtein ratio of over 0.85 or a Levenshtein distance of less than 2, we map it to the corresponding drug name in the drug dictionary. Note that each RxNorm medication has a unique identifier, known as an Rx Concept Unique Identifier (RxCUI).

**Fig. 2:**
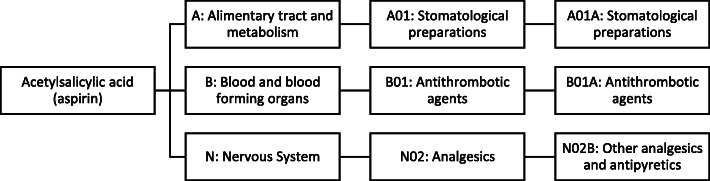
An example of ATC codes (level 1–3) for medication acetylsalicylic acid (aspirin)

The RxNorm REST API allows for us to easily map medication names to classification systems for different hierarchies of medication classes, via the RxCUI. In our framework, we used the REST API to search for medication RxCUI identification numbers from the RxNorm database after the RxNorm name was matched to the raw unstructured text. Afterwards we use the RxCUI number to map the matched medication name to any number of additional known hierarchies. Two examples of medication hierarchies include the ATC classification system, and the Medical Subject Headings Pharmacological Actions (MeSHPA) hierarchy. Due to slight differences in how different medication hierarchies are structured, the specific choice of medication hierarchy to use is dependent upon the goals of the researcher. An example of the medication term hierarchy from the ATC classification system is shown in Fig. 2.

#### Processing and mapping of reasons for medication use

**Fig. 3:**
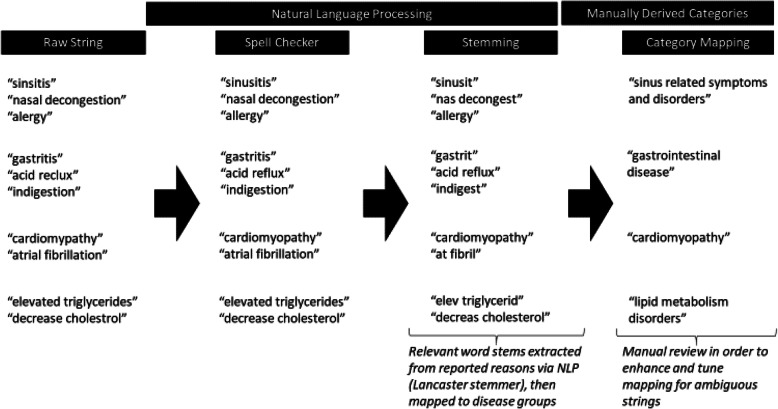
Examples of conditions/symptoms in the unstructured text and eventual mapping

We map the reasons for taking medications (conditions or symptoms) into categories via a natural language processing protocol that contains two main components: auto-spell correction and stemming. After the reasons are processed into word stems, we map the word stems into disease categories based on organ system involved. Examples of these reasons and their final intended mappings are shown in Fig. 3.

Similar to the processing of medication names, the auto-spell correction for reasons is conducted by comparing individual words against a dictionary (Python NLTK) and transforming a misspelled word to the dictionary word with highest Levenshtein ratio that is greater than 0.85. Afterwards, the word is further processed via stemming and category mapping.

The next step in mapping reasons to disease classes is to extract word stems from all reasons in all records using the Lancaster Stemmer. Word stems are smaller subparts of words, which are often found repeatedly in a wide variety of whole words. For example, the stem “arthr” refers to the concept of joints. Examples of words that have “arthr” as a stem include “osteoarthritis”, “rheumatoid arthritis”, and “arthroplasty”. We used the python package Natural Language Toolkit [12], which contains a module for extraction with Lancaster stemmer.

After extracting the word stems corresponding to the reasons, we manually create a map of common stems to broader categories of reasons. For example, if the original reason was the string “ulcerative colitis”, the Lancaster stemmer would extract the stem “ulc colit”, and we would manually map the reason category was “gastrointestinal disorders”. We store this mapping in a dictionary, a data structure that maps lookup values (“keys”) to results (“values”). The dictionary allows us to look up reason categories for any given word stem (i.e., looking up the stem “ulc colit” would return the reason “gastrointestinal disorders”. After creating the initial mapping of stems to classes, we use the Lancaster stemmer to extract stems from each of the reasons in each data record. For each reason in each record, we compare the stems to each of the keys in the dictionary. If the reason associated with any given key closely matches the raw reason string, we match the raw reason to the reason category in the dictionary. We match the raw reason to a dictionary key (e.g., word stems) if there is a Levenshtein distance of less than 2, and a Levenshtein ratio of over 0.85 between the raw reason stem and the stems in the dictionary. In the final implementation of the automation framework, we determined the thresholds for Levenshtein distance and ratio experimentally by manually trying different cutoffs of Levenshtein distance and Levenshtein ratio that led to 85% of the matches being made correctly. After this matching process, we manually corrected any remaining obvious errors (e.g., a raw reason mapping to an incorrect reason category due to misspelling of the reason) (see examples in Table 1).

**Table 1:** Example of reason mapping from original unstructured text to a reason category

Original Word	Stems	Reason Classes
arthritis	arthr	arthritis
musculoskeletal	musculoskelet	joint/musculoskeletal problem
gastritis	gastrit	gastrointestinal disease
ulcerative colitis	ulc colit	gastrointestinal disease
asthmatic	asthm	asthma
diarrhea	diarrhe	autonomic symptoms
depression	depress	depression and related disorders
mood	anxy	anxiety disorders
diabetes	diabet	diabetes

As medications are typically prescribed by providers and since they typically can be mapped consistently to an established hierarchy such as the ATC classification system, the majority of manual curation occurs in the reason mapping step.

After concepts for medications and conditions or symptoms for medication use are extracted from the unstructured text, they may be transformed for use in subsequent data analysis. For example, the medication and condition classes can be used in analytical studies as characterization or subtyping of diseases, as well as predictive modeling studies using machine learning algorithms that use the medication and condition classes as predictive features.

### Data structure and data sources

Any form of unstructured text that contains information for medications and reasons for medication usage (e.g., conditions or symptoms) can be used as input into our automation framework. Initial raw medications and reasons are stored as unstructured text, and typically contain spelling errors, which were variable across participants. In Table 2, we show examples of medications intended to be recorded, alongside their varied representations. Note that there may be large variance in how these are represented due to usage of brand names, differences in naming conventions between providers, or errors in spelling. Furthermore, since medications from epidemiological studies rely on self-reported data, they often include supplements taken by study subjects. Often times, these supplement names should be removed if the data are to be used for a subsequent analytical study.

**Table 2:** Examples of representations of medications and reasons captured in unstructured text from a public health setting

**Medications**	**Example representations**
Aspirin	Aspirin ASA, Aspirin Bayer, Aspirin Generic, ASA
Albuterol	Albuteral, Albuteral Nebulizer, Albuteral Inhaler
Metoprolol	Metoprold ER, Metoprolo ER
**Reason**	**Example representations**
Hypertension	High blood pressure, HTN, b/p
Arthritis	Arthritis (r) hip, arthritic joints, arthritis-anti-inflammatory

To show the utility of the proposed automation framework, we demonstrated its application on two data sources for Mylagic Encephalomyelitis/Chronic Fatigue Syndrome (ME/CFS): tertiary-based and population-based samples of 378 and 664 participants, respectively. The tertiary-based sample came from Stage-1 of the Multi-site Clinical Assessment of Myalgic Encephalomyelitis/Chronic Fatigue Syndrome (MCAM) study across multiple specialty clinics in USA [13]. The data for the population-based sample came from the follow-up phase of the population-based study in Georgia, USA including CFS, unwell and non-fatigued healthy controls [14]. Both source studies were approved by the Institutional Review Boards (IRB) of the Centers for Disease Control and local IRBs for field work. Assessment of medication information was one of the objectives for both source studies. We used a standardized abstraction form to record the medication information on medication names and reasons for medication use. In both of these studies, medication names and reasons for taking them were recorded as free-form text during the data collection process. For example, a participant may have reported taking the drug *metoprolol* for the reason of *hypertension*. The raw text collected from the abstraction form for these two concepts may have been exhibited as *“Metorpolo ER”* for metoprolol and *“high blood pressure”* for hypertension (see Table 2 for more examples). The raw text for all medications and reasons for all participants in these studies were used in the analysis. Please note that while other information about study participants were collected as part of the source studies, we only focused our analysis on the medications and reasons.”

## Results

The tertiary-based sample contained a total of 4858 raw records for medication information on 378 study subjects with CFS. After removing records corresponding to supplements, there were 2500 records remaining, each of which contains a raw medication and reason for taking the medication. Of these, there were 802 distinct raw medications and 596 distinct raw reasons. Using the automation framework, the distinct medications were automatically mapped into *65* categories according to the ATC classification system. After automated mapping, 54% of the distinct reasons required manual mapping. In total, the automatically and manually mapped reasons were condensed into *54* categories.

The population-based sample contained a total of 3823 raw records for medication information on 664 study subjects who were classified into CFS, insufficiently fatigued (ISF), and non-fatigued (NF). The subjects’ statuses were confirmed with a one-day clinic examination. All medications taken routinely or in the last 2 weeks of the clinic visit were recorded including medication name, dosage, mode, frequency, duration and reason for taking medications. After removing records corresponding to supplements via the supplement checker, there were 2810 records remaining. Of these, there were 907 distinct raw medications and 888 distinct raw reasons. Using the automation framework, the distinct medications were automatically mapped into *59* categories according to the ATC classification system. After automated mapping, 34% of the distinct reasons required manual mapping. In total, the automatically and manually mapped reasons were condensed into *91* categories (Table 3).

**Table 3:** Summary of feature set sizes for medications and reasons after the data processing workflow is applied

	# unique occurrences	
	Population based	Tertiary based
**Participants**	664	378
CFS	140	378
ISF	308	N/A
NF	216	N/A
**Medications (raw)**	907	802
after mapping	59	65
**Reasons (raw)**	888	596
after mapping	91	54

**Fig. 4:**
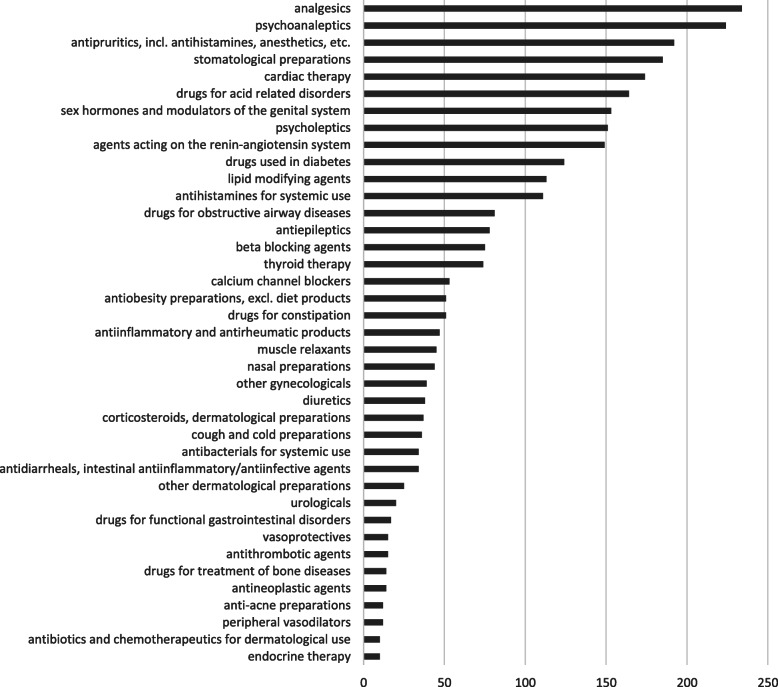
Most common medication categories after the data processing workflow is applied. The horizontal axis represents the number of records of the medication (across all patients; a patient can have multiple records)

**Fig. 5:**
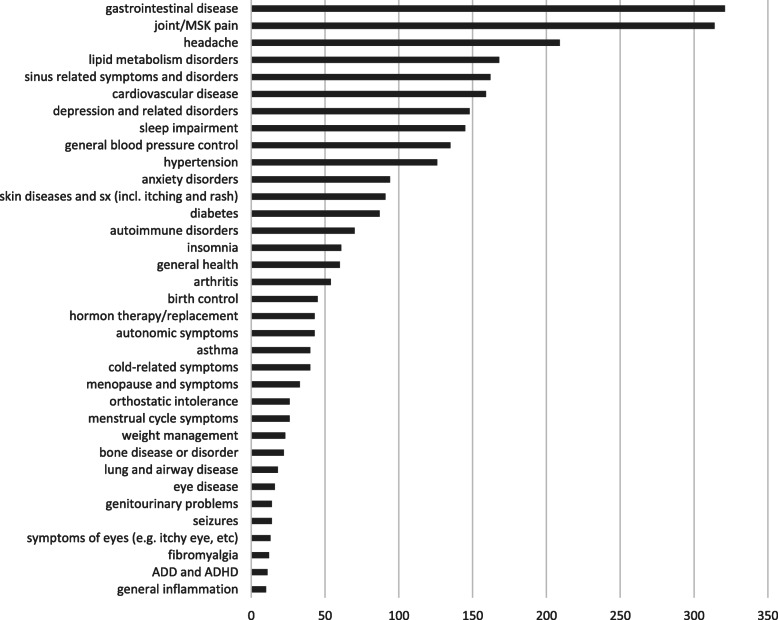
Most common reason (co-morbidity) categories after the data processing workflow is applied. The horizontal axis represents the number of records of the medication (across all patients; a patient can have multiple records)

We mapped medication names into the ATC classification system. The ATC system includes drug classifications at 5 levels: 1) anatomical main group; 2) therapeutic main group; 3) therapeutic/pharmacologic subgroup; 4) chemical/therapeutic/pharmacological subgroup; and 5) chemical substance. We used the RxNorm REST API to correct any obvious misspellings, map the medication field to an RxCUI, and then obtain the ATC codes for the drugs up to the 3rd level, which represents the therapeutic class of the drug. Note that one drug can map to multiple ATC codes under this mapping. An example for the medication mapping is shown in Fig. 2. Examples of supplements excluded, as well as misspellings are shown in Table 4. Note that the RxNorm REST API allowed us to differentiate supplements from drugs. For this particular demonstration we excluded supplements and only kept drugs for ATC therapeutic class. An illustration of the most prevalent medications after mapping, are shown in Fig. 4. An illustration of the most prevalent reasons after mapping, are shown in Fig. 5.

**Table 4:** Example of original representations of medication names, potential alternate representation (e.g., misspelling) which are corrected by the automation framework, and the final medication class assigned via the ATC system

Medication Name	Potential Alternate Representation	Final Medication Class
Fexofenadine	Fexafenedine, fexfenadine	antihistamines
Hydrochlorothiazide	Hydrochlorothazide, Hydrochlorthiazide	diuretics
Metoprolol	Metoprolol Succ ER, Metoprolol Tart	beta-adrenergic blocking agents
Quinapril	Quinipril	angiotensin converting enzyme inhibitors
Vitamin B12	Vit B12, vitemin B12	supplement - excluded

To demonstrate the time requirement difference, we measured the amount of time in seconds taken to perform an extraction manually for 10 different records, and compared the average time taken to extract medication information per record when performed manually, to the average time taken to extract the information when using the automation framework. In the manual method, the raw medication data was viewed in unstructured text, typos were corrected and the specific drug name was queried in the ATC database. The class of medication was obtained manually from the database. When performed manually, medication records took approximately 22 s to parse and look up the medication class. When performed with the automation framework, a medication record took approximately 2 s to process and convert to a medication class. This represents an approximately 91% reduction in time taken to process the unstructured text and obtain a meaningful medication feature for use in future data analytics processes.

The automation framework resulted in a significant reduction in time taken to perform mapping of raw medications and reasons into higher-level, interpretable categories. After removing supplements, the majority of medications were able to be automatically mapped into the ATC hierarchy. Combined across both study samples, there were a total of 1266 unique drugs, of which 1068 were able to be automatically mapped into 89 distinct drug categories. This represents an 84.4% reduction in the manual processing that would otherwise be required to map all drugs into categories. However, mapping of reasons required more manual intervention. Combined across both study samples, there were a total of 1432 distinct reasons for medication use, of which 850 were automatically mapped into 65 distinct reason categories. This represents a 59.4% reduction in the manual processing that would otherwise be required to map all reasons into categories.

## Discussion

The workflow for mapping medications and reasons which we have proposed is a process that can be re-used in different scenarios where medication names and reasons are stored as unstructured text. Our technique allows for an automated process of extracting medication and reason names and subsequently mapping them to higher order classes. The process can be tuned depending on the desired level of granularity of medication and reason features. It is important to note that in our specific implementation, manual curation of the reasons for medication usage was used as a follow-on process after the automation framework was run. For the population-based sample, there were 194 words generated from the stemming process (compared to 888 originally), which were eventually manually curated into 91 different categories. For the tertiary-based sample, there were 145 words generated from the stemming process (compared to 596 originally), which were eventually manually curated into 54 categories. Nonetheless, our automation framework was able to remove substantial human supervision that would have otherwise been required had NLP not been used to gather word stems. Our automation framework is instrumental for reducing the time required to extract features from unstructured text: about 91% reduction in time taken to process the unstructured text and obtain a meaningful medication feature for use in future data analytics processes.

While we have demonstrated the data processing workflow on samples from ME/CFS studies, this method can be used on studies for any disease in which data are stored as unstructured text. Furthermore, this process can also be applied toward analysis of clinical notes, which are often stored as unstructured text in an unstructured format. It is important to note that our study was intended to be an illustration of the general framework for processing free form raw text representing medications and reasons, rather than a study intended to draw clinical conclusions about ME/CFS. For that reason, we did not specifically choose a levenshtein threshold that was tuned to representative data but rather used a common, widely used default threshold. For future studies that involve downstream hypothesis testing with the extracted concepts, careful calibration of the Levenshtein threshold should be performed and a threshold that is tuned to a desired balance of precision and recall should be chosen. Such analysis should be performed using a separate dataset (i.e., of raw medication and reason strings) representative of the study dataset.

Using the resulting mappings of medications and reasons, subsequent data analysis including machine learning methods can be performed. For example, the medication and reason features can be used in predictive models for disease. Alternatively, the features can be used in unsupervised learning methods to discover subtypes of disease. Our framework was able to automatically map 84.4% for medications and 59.4% for reasons for medication use. The raw medications and reasons that were unable to mapped automatically typically were significant misspellings or uncommon representations of concepts (e.g., uncommon or incorrect abbreviations, unconventional description of a reason). In such cases, these terms that require manual mapping may have a higher level of difficulty for a human to map, compared to terms that were able to be mapped automatically.

There are many avenues for future work. Future research should address problems specific to clinical domains. For example, the word stemming could be customized to specific diseases of interest. In our particular use case, one possible improvement is to devise a customized stemming method that leverages a matching algorithm that matches stems for concepts highly relevant to symptoms that may be experienced by patients with ME/CFS. Such stems could be derived in a customized manner by a clinician or relevant domain expert. It is important to note that there are other stemming methods which could be used, including the Porter and Snowball (Porter2) methods. The Lancaster stemmer is more aggressive than the Porter stemmers, resulting in greater distinction between resulting stemmed words which allows for more distinctive automated concept mapping. However, the typically shorter stems resulting from Lancaster stemmer may be less readily intuitive to a human reader during the manual mapping phase. In future work, more in depth analysis of different stemming methods can be considered.

Additionally, improvements can be made to the manual intervention involved in processing medications and reasons. For example, to reduce the manual time involved a computational method could be devised that compares the stemmed reasons against concepts in medical ontologies such as the Unified Medical Language System [15]. It is important to note that there does not exist a standard widely used methodology for this, and that careful consideration should be made of the trade-off between potential time savings and potential errors from mis-grouped concepts.

Furthermore, extraction and grouping of concepts could be performed on different data domains, such as unstructured data for procedures, medical symptoms, or behavioral properties of individual patients. Public data source, such as the National Ambulatory Medical Care Survey (NAMCS) data [16], could potentially leverage our natural language processing framework.

Our study provides a valuable framework for analyzing unstructured text, which is commonly excluded from analysis in many research studies involving machine learning on healthcare information. In traditional studies involving structured data sources such as electronic health record data, medication and diagnosis names are often stored in structured format and are often already recorded as a mapped category. For example, diagnoses are often encoded following the International Classification of Diseases (ICD-10) classification and medications are often encoded following the RxNorm classification in an electronic health record. Our study introduces a method that may allow researchers to leverage unstructured text and extract features for medications and reasons that are as useful as those features which may be obtained in structured data stores such as electronic health records. Our study should motivate future work in automating feature extraction from unstructured medical text data, especially for targeted domain-specific applications such as ME/CFS.

## Conclusions

Our proposed automation framework demonstrates the usefulness of NLP strategies even when there is no established mapping database. For less established knowledge data sources (e.g., reasons for medication use), the method is easily modifiable as new knowledge sources for mapping are introduced. The ability to condense large features into interpretable ones will be valuable for subsequent analytical studies involving techniques such as machine learning and data mining. Our automation framework via NLP also increase the completeness, timeliness, and accuracy of data while reducing the level of human (manual) intervention needed to identify critical data in narrative text. While our proposed automation framework demonstrated by the medication information collected from tertiary-based and population-based samples of ME/CFS, this framework can be applied to unstructured text medication information collected for patients with other illnesses or diseases a well.

## Supplementary information

**Additional file 1 Appendix Figure** A.1. An end-to-end illustration of each module of the medication processing (A) and reason processing (B) algorithms. For each type of input, raw strings are provided as input to the algorithm, and subsequently transformed through successive modules. The final result is a string representing the category. Each module is labeled accordingly in the code, provided at https://github.com/rchen25/medication_natural_language_processing.

## Data Availability

Restrictions by the data custodians mean that the datasets are not publicly available or able to be provided by the authors. The program codes used in the current study are available from the corresponding author on reasonable request. Researchers wanting to access the datasets used in this study should email CDC’s ME/CFS Program (cfs@cdc.gov) and discuss next steps for the data request. The ME/CFS program data review committee will grant the access after the review and the data use agreement is finalized.

## Abbreviations

ATC: Anatomical therapeutic chemical
ME/CFS: Mylagic encephalomyelitis/chronic fatigue syndrome
CDC: Centers for Disease Control and Prevention; Processing
CRIS-CODE: Clinical record interactive search comprehensive data extraction
cTAKES: Apache clinical text analysis and knowledge extraction system
CUI: Concept unique identifier
ICD: International Classification of Diseases
IRB: Institutional Review Board
ISF: Insufficiently fatigued
MeSHPA: Medical Subject Headings Pharmacological Actions
NAMCS: National Ambulatory Medical Care Survey
NF: Non-fatigued
NLP: Natural language
SNOMED: Systematized nomenclature of medicine
REST API: Representational state transfer application programming interface
WHO: World Health Organization
USA: United States of America
