# Supplementary figures and images for: Extracting medication information from unstructured public health data: a demonstration on data from population-based and tertiary-based samples
Source: BMC Med Res Methodol. 2020 Oct 15;20:258. doi: 10.1186/s12874-020-01131-7 (PMC7559204; doi:10.1186/s12874-020-01131-7)

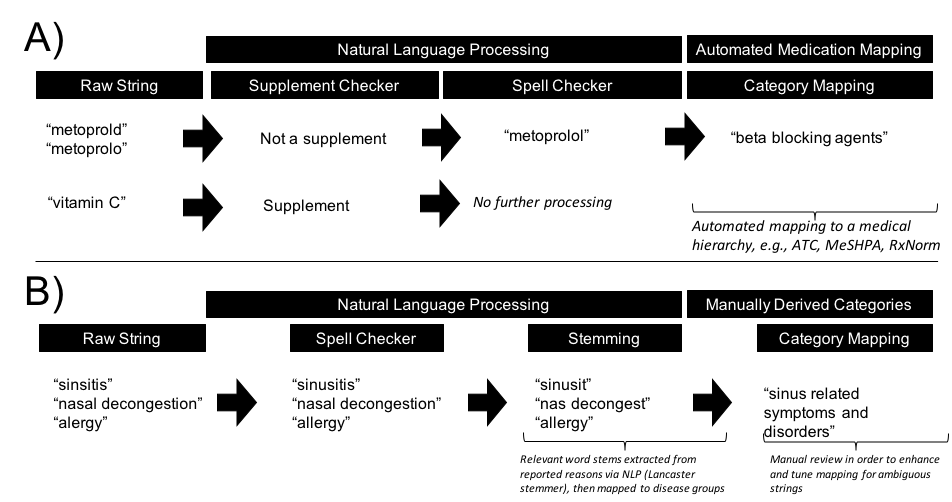

Supplement: Supplementary file 1 — Additional file 1 Appendix Figure A.1. An end-to-end illustration of each module of the medication processing (A) and reason processing (B) algorithms. For each type of input, raw strings are provided as input to the algorithm, and subsequently transformed through successive modules. The final result is a string representing the category. Each module is labeled accordingly in the code, provided at https://github.com/rchen25/medication_natural_language_processing. [file 12874_2020_1131_MOESM1_ESM.png]
